# The haves and the have‐nots: Identifying typologies of change in relative deprivation using multi‐trajectory latent class growth analysis

**DOI:** 10.1111/bjso.70009

**Published:** 2025-08-16

**Authors:** Kieren J. Lilly, Chris G. Sibley, Danny Osborne

**Affiliations:** ^1^ Institute for Social Science Research University of Queensland Brisbane Australia; ^2^ School of Psychology University of Auckland Auckland New Zealand

**Keywords:** inequality, latent class growth analysis, mixture modelling, relative deprivation

## Abstract

Relative deprivation theory argues that individuals can perceive themselves to be deprived relative to other individuals (IRD) or that their ingroup is deprived relative to other groups (GRD). Few studies, however, investigate how these distinct ‘types’ of relative deprivation manifest over time. We address this oversight using multi‐trajectory latent class growth analysis to identify distinct growth trajectories of relative deprivation across 13 annual waves of a nationwide longitudinal panel study (2011–2023; *N*_total_ = 75,073). We identified two discrete classes: the Content class (90.5% of the sample) and the Deprived class (9.5%). Whereas the Content class had low levels of IRD and GRD that declined over 12 years, the Deprived class had moderate levels of IRD that decreased but high levels of GRD that *increased* over time. Membership in these two classes differed across demographics, well‐being and sociopolitical measures. The implications for relative deprivation theory are discussed.

## INTRODUCTION

A house may be large or small; as long as the neighbouring houses are likewise small, it satisfies all social requirements for a residence. But let there arise next to the little house a palace, and the little house shrinks into a hut. The little house now makes it clear that its inmate has no social position at all to maintain. Marx (1847/1935, p. 33)

Marx (1847/1935) persuasively argues that one's objective position in society is less indicative of how they will respond than how they *perceive* their status relative to others. Indeed, research reveals strong associations between perceived relative deprivation and poor physical (Adler et al., 2000; Beshai et al., 2017) and mental health (Lyu & Sun, 2020; Schmitt et al., 2010; Smith et al., 2020), as well as intergroup prejudice (Guimond & Dambrun, 2002; Pettigrew et al., 2008) and collective action (Abrams & Grant, 2012). Moreover, the effects of fiscal relative deprivation on these outcomes are generally larger than those of *objective* indicators like income (Smith et al., 2012). Thus, relative deprivation is crucial for understanding how people respond to disadvantage.

Importantly, different *types* of relative deprivation predict distinct responses (Runciman, 1966; Smith & Huo, 2014). Runciman (1966) distinguished between people who perceive themselves to be deprived relative to other individuals (individual‐based relative deprivation; IRD) and people who perceive their ingroup to be deprived relative to other groups (group‐based relative deprivation; GRD). Because these discrete constructs stem from different comparison processes (Crosby, 1976; Smith et al., 2012), people may perceive their personal and group status differently. Specifically, people can have (a) low IRD and GRD, (b) high IRD but low GRD, (c) low IRD but high GRD and (d) high IRD and GRD (Foster & Matheson, 1995; Runciman, 1966).

Although research assumes different ‘types’ of relative deprivation exist, few studies examine these typologies in the population (for an exception, see Osborne et al., 2015). Thus, the extent to which these profiles exist—and for whom—is mostly unexplored. Moreover, few studies examine how (and for whom) feelings of relative deprivation *change* over time. Identifying these processes would increase understanding of how different people experience relative deprivation and for whom these feelings are most prevalent.

The current research addresses these oversights by examining feelings of fiscal relative deprivation among a nationwide random sample of New Zealand adults over 12 years (2011–2023). Specifically, we utilize multi‐trajectory latent class growth modelling (LCGA) to identify different latent groups of people based on how their feelings of IRD *and* GRD change over time. After establishing these latent classes, we examine the demographic correlates of class membership. Finally, we use membership in these distinct latent classes to predict differences in well‐being, sociopolitical attitudes and study retention, allowing us to identify how different trajectories of relative deprivation may impact both individuals and society. We begin by reviewing the relevant relative deprivation literature before outlining how New Zealand's socio‐economic context may influence changes in relative deprivation. We then introduce the strengths of LCGA for modelling longitudinal change and conclude with an overview of our study.

### Relative deprivation theory

Beginning with Stouffer et al.'s (1949) seminal work, social scientists define relative deprivation as perceptions of disadvantage relative to (similar) others. Because social comparisons form the basis of these perceptions (see Pettigrew, 1967), different comparison targets produce distinct feelings of relative deprivation. Indeed, Runciman (1966) noted a key distinction between egoistic (individual) and fraternal (group) deprivation. Specifically, IRD stems from individuals comparing *themselves* to other similar individuals, while GRD stems from individuals comparing their *ingroup* to other similar groups. Accordingly, IRD (relative to GRD) is more strongly associated with individual‐based outcomes, including reduced physical and mental health (Beshai et al., 2017; Callan et al., 2015; Schmitt et al., 2010). Conversely, GRD (relative to IRD) strongly correlates with group‐based outcomes, including protest support (Abrams & Grant, 2012; Grant & Brown, 1995) and intergroup prejudice (Guimond & Dambrun, 2002; Pettigrew et al., 2008).

Because these discrete constructs stem from different comparison processes, one can feel personally deprived but collectively advantaged (or vice versa). Accordingly, Runciman (1966) argued for four ‘categories’ of relative deprivation. The first, coined *orthodox*, refers to individuals low on IRD and GRD who are content with their status. Conversely, *strivers* score high on IRD but low on GRD, reflecting their frustration towards their *personal* (rather than collective) position, whereas *fraternalists* score low on IRD but high on GRD. Finally, the *doubly deprived* are dissatisfied with their individual *and* ingroup position and, thus, are high on IRD and GRD. Runciman argued that the latter group is ‘the most relatively deprived of all’ (p. 34) and, therefore, particularly motivated to engage in collective action to redress their disadvantage (Runciman, 1966).

Although Runciman's (1966) relative deprivation framework is enduring, few studies have examined these categories in practice, and fewer find evidence of the doubly deprived. While early studies suggest that IRD and GRD have additive effects (Dibble, 1981; Foster & Matheson, 1995; Smith et al., 1994), more recent research fails to uncover evidence of double deprivation in the general population (see Osborne et al., 2015; Pettigrew et al., 2008). Rather, people who perceive their *group* as disadvantaged are unlikely to perceive *themselves* as such (Crosby, 1984). Indeed, people often perceive more discrimination directed at their group than at themselves as *members* of that group (Taylor et al., 1990). This personal/group discrepancy is readily apparent among objectively disadvantaged groups where group‐based discrimination is particularly salient (e.g. McAvay & Safi, 2025; Taylor et al., 1996). Thus, when one's group membership is made salient by disadvantage, intergroup comparisons are more likely than interpersonal comparisons (Dumont et al., 2006). Accordingly, people are unlikely to perceive both themselves and their group as ‘equally’ deprived.

Research also suggests that feelings of IRD may be an *antecedent* to GRD (Lilly et al., 2023; Pettigrew, 2002; Tougas & Beaton, 2002). Indeed, some studies find a ‘spillover’ effect whereby GRD mediates the relationship between IRD and prejudice (e.g. Pettigrew et al., 2008; Tougas & Beaton, 2002). Likewise, more recent research suggests that within‐person changes in IRD predict changes in GRD (Lilly et al., 2023). Rather than ‘double deprivation’ whereby people feel personally and collectively deprived *simultaneously*, IRD may foster GRD (though high IRD is not a necessary condition for high GRD; Pettigrew, 2002).

Collectively, these findings challenge Runciman's (1966) typology and demonstrate the need to reframe our understanding of the relationship between IRD and GRD. That said, this typology is vastly understudied (but see Osborne et al., 2015), and thus, ‘types’ of relative deprivation and their prevalence in the population require further investigation. Perhaps more importantly, longitudinal research is needed to identify how feelings of relative deprivation may develop and change differently among different groups.

### Relative deprivation in New Zealand

The current study focuses on fiscal measures of relative deprivation (i.e. feeling *financially* deprived relative to other individuals or groups) in New Zealand, with our measure of fiscal GRD focusing on ethnicity. The marked socio‐economic shifts throughout the 21st century, particularly during our assessment period (2011–2023), illustrate the need to investigate these relative deprivation domains over time. Indeed, like many nations, the 2007–2010 global financial crisis deleteriously impacted New Zealand's Gross Domestic Product, unemployment rates, and cost of living (e.g. Mare, 2022; Mils, 2010). These financial pressures increased alongside rising *wealth* among society's elite (see Atkinson et al., 2011; Peters & Wilson, 2008), fostering ill‐being and discontent among the general population in New Zealand (Sibley et al., 2011) and internationally (e.g. Ragnarsdóttir et al., 2013). This discontent culminated in the 2011 Occupy Movement, which saw protests over the growing divide between the poor and the wealthy across 80+ nations, including New Zealand (Hager, 2021; Rogers, 2012). The movement was short‐lived; however, economic inequalities grew throughout the mid‐2010s (Stephens & Cleveland, 2024). Our earliest assessments thus capture relative deprivation during a time of considerable economic strain and discontent in New Zealand.

Income inequality has since declined in New Zealand, and today the nation is relatively low‐to‐moderate in poverty and inequality compared to other Western nations (e.g. the United States; see OECD, 2023). That said, New Zealand's economic systems continue to privilege the wealthy (Rashbrooke, 2013, 2021); the nation is ranked 136th out of 161 countries for fair wealth distribution (Oxfam Aotearoa, 2023) and 16th out of 146 countries for cost of living (Numbeo, 2024). Moreover, the economic fallout from the pandemic (e.g. Choi et al., 2021; Fletcher et al., 2022; Howard et al., 2022) and the most recent recession (McIlraith, 2025) marks renewed socio‐economic pressures for New Zealand. Such pressures are made more salient by the intersections of income and racial inequalities, with ethnic minorities disproportionately affected by socio‐economic crises and poverty internationally (Iceland, 2019; Pager & Shepherd, 2008) and nationally (Easton, 2013; Marriott & Alinaghi, 2021; Sibley et al., 2011). Thus, although New Zealand has an egalitarian reputation, socio‐economic crises have waxed and waned over the past two decades, leaving some more disadvantaged than others (for discussion, see Rashbrooke, 2021).

Against this backdrop of socio‐economic change, changes in fiscal (and ethnic) relative deprivation likely occurred over time. Indeed, economic events such as global recessions and pandemics foster both IRD (e.g. Kiebler & Stewart, 2021; Ragnarsdóttir et al., 2013) and GRD (particularly among ethnic minorities; Lilly et al., 2024b). Prior longitudinal work using our dataset also suggests that relative deprivation changes as a function of group status, normative ageing and the unique experiences of younger generations (Lilly et al., 2025), as well as within‐person changes in income (Lilly et al., 2024a). Beyond New Zealand, research by de la Sablonnière and colleagues highlights how (perceived) negative societal changes influence relative deprivation in nations across cultures (de la Sablonnière, Taylor, et al., 2009; de la Sablonnière & Tougas, 2008; de la Sablonnière, Tougas, et al., 2009). Taken together, this research illustrates the importance of studying how feelings of relative deprivation change over time.

Although research identifies how changes in relative deprivation emerge across time and generations (e.g. Lilly et al., 2025), these analyses focus on understanding changes in IRD and GRD *independently*. Such analyses do not account for potential combinations of IRD and GRD, leaving questions as to how and when people experience different forms of relative deprivation simultaneously. Likewise, research generally focuses on trends across an entire sample, which may occlude distinct subsets of the population who differ in their relative deprivation scores over time. For instance, the rarity of income‐focused collective action in New Zealand suggests that many feel content with their societal position (for discussion, see Osborne & Sibley, 2013). Others, however, may feel increasingly discontent over time, particularly after major socio‐economic crises. Examining these nuances will increase understanding of when people respond to perceived disadvantage—and when they may not.

### Latent class growth analysis

LCGA is a modelling technique that identifies distinct subsets of the population that differ in their rates of change in a construct over time (Muthén, 2004; Muthén & Muthén, 2000). Critically, LCGA assumes that all growth trajectories within a class are homogenous and thus constrains the growth factor variances and covariances to zero. Therefore, any individual differences are assumed to exist only *between* (rather than within) classes (Jung & Wickrama, 2008). In doing so, LCGA accounts for the unobserved heterogeneity between latent classes within a population.

LCGA has been extensively developed by Nagin and colleagues (e.g. see Nagin, 1999, 2005; Nagin & Land, 1993) and is generally preferred to conventional growth modelling when examining trajectories of health behaviours or medical disorders (e.g. Downie et al., 2016; Hockenberry et al., 2017; Hodgekins et al., 2015). However, LCGA remains relatively underutilized in studies of inequality and discrimination, despite its potential to uncover unique trajectories in psychological constructs over time (see Thomas et al., 2024). In the present study, LCGA is ideally suited to examine ‘types’' of relative deprivation across time. Specifically, its ability to identify how different subgroups in the population differ in their feelings of relative deprivation longitudinally allows us to test the extent to which Runciman's (1966) framework (see also Foster & Matheson, 1995) and the personal/group discrimination discrepancy (Crosby, 1984; Taylor et al., 1990) account for how people perceive their societal position over time.

### Overview of the current study

The current study utilizes LCGA to examine feelings of relative deprivation across 12 years of data from a large, nationwide random sample of adults. Critically, we estimate multiple‐trajectory classes based on the trajectories of IRD *and* GRD, allowing for distinct ‘types’ of relative deprivation akin to those posed by Runciman (1966) to emerge. The scale of our data, coupled with the ability of LCGA to identify distinct classes of people, provides a robust test of this oft‐discussed typology while also considering that feelings of IRD and GRD may change over time. Given that both typologies of relative deprivation and its development over time are vastly understudied, the current research extends the extant literature by identifying how, when and for whom these feelings are most prevalent.

Given the lack of research examining typologies of relative deprivation, we make no specific predictions as to the number of trajectory classes nor their rates of change over time. However, we consider the possibilities proposed by Runciman (1966), results from past cross‐sectional research (Osborne et al., 2015) and broad socio‐economic trends in New Zealand over our assessment period. Namely, we expect to identify a substantial proportion of the population that is generally low and stable on both IRD and GRD, given that most of the population is likely to be relatively content with their societal position (see Osborne et al., 2015). Conversely, any subgroup of the population that scores high or is increasing in IRD or GRD should be a relatively *small* percentage of the sample. Finally, we expect a ‘doubly‐deprived’ subgroup with comparably high levels of IRD and GRD over time to be trivial or absent from the population, given the lack of empirical support for double deprivation (Osborne et al., 2015; Pettigrew et al., 2008) and considerable evidence of a personal/group discrimination discrepancy (see Crosby, 1984; McAvay & Safi, 2025; Taylor et al., 1990). After identifying the classes, we examine (a) the demographic predictors of class membership and (b) mean differences in well‐being, institutional trust and satisfaction, support for social change and study retention across classes. In doing so, we aim to identify qualitative differences between our estimated classes and, thus, distinct subgroups of the population who experience unique levels of relative deprivation across time.

## METHOD

### Sampling procedure and participants

We use data from the New Zealand Attitudes and Values Study (NZAVS)—a nationwide random probability sample of adults that began in 2009. The NZAVS was approved by the University of Auckland Human Participants Ethics Committee, and informed consent was obtained from participants. Participants of the NZAVS were initially sampled from the electoral roll, which is compulsory in New Zealand (Time 1; *N* = 6518, response rate 16.6%). Eight subsequent booster samples were conducted at Times 3, 4, 5, 8, 10, 11, 14 and 15 to address sample attrition and diversify the sample. By Time 15, 76,409 participants had completed at least one wave of the study. Sibley (2023) provides further information on the sampling procedure, retention rates and ethics approvals for the NZAVS.

Although the NZAVS began in 2009, we first measured relative deprivation at Time 3 in 2011. Accordingly, we focus on participants who completed at least one wave of the study from Time 3 (2011) to Time 15 (2023; *N*_total_ = 75,073; *M*_waves_ = 4.48; *SD* = 3.13). Of these participants, 49,454 (65.9%) completed three or more waves, and 1,598 (3.1%) completed all 13 assessments. The proportion of complete cases on a single or pair of variables ranged from 0.04 to 0.64 (*M* = 0.18, *SD* = 0.12). Accordingly, we utilized full‐information maximum likelihood estimation (FIML) to allow the inclusion of both partial and complete responses in our analyses (Enders & Bandalos, 2001).

Of our 75,073 participants, 62.1% were women, 36.8% were men and 1.1% were gender diverse. Most participants were born in New Zealand (77.3%), and the average age of participants in 2011 (Time 3) was 39.90 (*SD* = 15.98). In terms of ethnicity, most participants identified as New Zealand European (77.8%) or Māori (13.2%), with the remainder identifying as Asian (6.2%) or Pasifika (2.8%). Table 1 provides further demographic information for participants at each wave.

**TABLE 1:** Sample demographic information at each wave of our analyses.

Variables	Time 3	Time 4	Time 5	Time 6	Time 7	Time 8	Time 9	Time 10	Time 11	Time 12	Time 13	Time 14	Time 15
Gender^a^ (%)													
Women	62.5	62.5	62.8	63.2	62.6	62.6	63.3	62.6	63.8	63.6	63.9	63.1	62.8
Men	37.5	37.5	37.2	36.7	37.3	37.3	36.6	37.2	35.8	35.8	35.5	36.0	36.2
Gender‐diverse	–	–	–	0.1	0.1	0.2	0.1	0.2	0.4	0.5	0.6	0.9	1.0
Age, *M* (*SD*)	50.44	49.10	47.64	49.33	50.80	49.62	51.33	48.59	51.56	52.95	54.39	53.62	52.78
	(15.95)	(15.03)	(14.08)	(14.04)	(13.90)	(13.93)	(13.77)	(13.86)	(13.87)	(13.69)	(13.66)	(15.70)	(16.77)
Born in NZ (%)	78.8	79.0	79.4	79.5	79.9	79.3	79.5	78.3	78.2	78.5	78.6	80.5	77.9
Ethnicity (%)													
European	80.8	73.7	79.3	80.5	81.5	81.7	82.3	82.8	83.7	85.4	86.1	85.5	83.5
Māori	12.6	17.1	13.4	12.6	12.3	11.6	11.9	10.1	10.3	8.9	9.1	9.4	9.8
Pasifika	2.5	4.3	3.0	2.8	2.6	2.3	1.9	1.9	2.0	1.9	1.5	1.5	1.6
Asian	4.0	4.8	4.4	4.1	3.6	4.3	3.9	5.2	4.1	3.8	3.4	3.6	5.2
Employed (%)	71.5	71.2	75.5	76.2	75.2	78.8	74.8	79.4	75.7	76.6	74.1	70.6	70.2
Homeowner (%)	–	–	–	–	76.9	–	–	73.3	–	79.7	–	76.8	73.9
Income^b^, *M* (*SD*)	0.91	0.96	1.02	1.05	1.07	1.09	1.14	1.15	1.18	1.21	1.25	1.29	1.30
	(0.74)	(0.81)	(0.82)	(0.88)	(0.87)	(0.92)	(0.94)	(0.92)	(1.08)	(1.05)	(1.11)	(1.41)	(1.21)
Sample size	6883	12,177	18,260	15,820	13,942	21,936	17,071	47,944	42,680	38,550	34,131	33,717	32,847

^a^ Gender was measured with a binary male/female question in Times 3–5, and an open‐ended measure from Time 6 onwards.

^b^ Annual household income (before tax) divided by NZD$100,000.

### Measures

Unless otherwise specified, items were measured at every assessment occasion (i.e. Time 3 to Time 15), rated on a 1 (*strongly disagree*) to 7 (*strongly agree*) scale and averaged to represent their respective constructs.

#### Relative deprivation

Our measures comprise two items independently assessing the cognitive and affective components of relative deprivation, consistent with best practice for relative deprivation measures (Smith et al., 2012). Individual‐based relative deprivation (IRD) was measured using two items adapted from Abrams and Grant (2012): (a) ‘I'm frustrated by what I earn relative to other people in New Zealand^1^’ (affective IRD), and (b) ‘I generally earn less than other people in New Zealand’ (cognitive IRD; *α*s = .54–.65). Group‐based relative deprivation (GRD) was also measured using two items adapted from Abrams and Grant (2012): (a) ‘I'm frustrated with what my ethnic group earns relative to other groups in New Zealand’ (affective GRD), and (b) ‘People from my ethnic group generally earn less than other groups in New Zealand’ (cognitive GRD; *α*s = .58–.63).

#### Predictors

We used gender, age, ethnicity, home ownership, whether participants were born in New Zealand, and participants' earliest reported household income and education level as demographic predictors in our analyses. Gender was dummy‐coded (0 = woman, 1 = man); gender‐diverse participants were included in our overall analysis but excluded from the gender variable due to sample size constraints. Age was measured using participants' age at Time 3. Ethnicity was measured by asking participants to select the ethnic group(s) to which they belong and then priority‐coding responses into four mutually exclusive ethnic groups (see Table 1 for a breakdown of these ethnic groups in each wave). Māori had priority over all other ethnicities, followed by ‘Pacific’, ‘Asian’ and then ‘European’, with those who did not fit into these four categories excluded from the variable. For our analyses, we used the European group as the reference category for three dummy‐coded variables capturing participants' ethnicity. Homeownership was measured at Times 7 (2015), 10 (2018), 12 (2020), 14 (2022) and 15 (2023) using the forced‐choice yes/no item: ‘Do you own your own home? (either partially or fully owned)’. We dummy‐coded participants' responses based on whether they had *ever* reported owning their home (0 = no, 1 = yes). Additionally, we dummy‐coded whether participants were born in New Zealand (0 = no, 1 = yes).

We assessed household income at each assessment by asking participants, ‘Please estimate your total household income (before tax) for the year’. We used each participant's first reported household income (divided by NZD$100,000) to maximize the number of unique cases used in our analysis. We measured education by using participants' first reported response to the item, ‘What is your highest level of qualification?’ Responses were coded into an 11‐level ordinal variable according to the New Zealand Qualifications Authority (0 = no formal qualification, 10 = doctoral degree or equivalent).

Finally, we assessed how strongly participants identified with their ethnicity using the mean of each participants' first response to three items from Leach et al. (2008): (a) ‘I often think about the fact that I am a member of my ethnic group’; (b) ‘The fact that I am a member of my ethnic group is an important part of my identity’; and (c) ‘Being a member of my ethnic group is an important part of how I see myself’ (*α*s = .77–.82).

#### Well‐being and sociopolitical attitudes

To maximize the number of unique cases for variables assessed at multiple waves, we assessed the following outcome variables using participants' *most recent* response. We rescaled all measures on a 0–1 scale to facilitate comparisons.

**Personal well‐being** was measured using the mean of four items from Cummins et al.'s (2003) Personal Wellbeing Index. Participants were asked to rate their satisfaction with their: (a) standard of living, (b) health, (c) future security and (d) personal relationships on a 0 (*completely dissatisfied*) to 10 (*completely satisfied*) scale (*α*s = .72–.76).

**Psychological distress** was measured using the mean of Kessler et al.'s (2010) K6 psychological distress scale. The scale contains six items measured on a 0 (*none of the time*) to 4 (*all of the time*) scale, with participants reporting how often they felt the following in the last 30 days: (a) ‘…hopeless?’; (b) ‘…so depressed that nothing could cheer you up?’; (c) ‘…restless or fidgety?’; (d) ‘…that everything was an effort?’; (e) ‘…worthless?’ and (f) ‘…nervous?’ (*α*s = .84–.87).

**National well‐being** was assessed using the mean of three items from Tiliouine et al. (2006). Participants were asked to rate their satisfaction with: (a) ‘The economic situation in New Zealand’; (b) ‘The social conditions in New Zealand’; and (c) ‘Business in New Zealand’ on a 0 (*completely dissatisfied*) to 10 (*completely satisfied*) scale (*α*s = .70–.83).

**National identification** was assessed at Times 7, 8 and 10 through 13 using a single item: ‘I identify with New Zealand’ (Postmes et al., 2013).

**Trust in politicians** was assessed at Times 9 through 15 using a single item: ‘Politicians in New Zealand can generally be trusted’ (Sibley et al., 2020).

**Satisfaction with government** was assessed using a single item. Participants were asked to rate their satisfaction with ‘The performance of the current New Zealand government’ on a 0 (*completely dissatisfied*) to 10 (*completely satisfied*) scale.

**General conspiracy beliefs** was assessed at Times 11 through 15 using a single item from Lantian et al. (2016): ‘I think that the official version of major world events given by authorities often hides the truth’.

**Support for income redistribution** was assessed at Times 6 through 11, and Times 14 and 15, using a single item adapted from Gallup Organisation (2008): ‘Redistributing money and wealth more evenly among a larger percentage of the people in New Zealand through heavy taxes on the rich’.

**Support for collective action** was measured at Times 5 through 15 using the mean of three items from Cronin et al. (2012): (a) ‘I have considered voting in terms of what is good for my particular ethnic group’; (b) ‘I have considered participating in demonstrations on behalf of my ethnic group’ and (c) ‘I have considered signing petitions on behalf of my ethnic group’ (*α*s = .76–.80).

#### Retention

In addition to our well‐being and sociopolitical outcomes, we examined whether the identified classes differed in the probability that they would complete the most recent wave used in our analyses (i.e. Time 15). The NZAVS forecasts retention using a neural network initially trained using 99,217 cases comprised of snapshots of the NZAVS dataset at Time 9 and Time 12 (Sibley, 2023, presents a full model overview). The model estimates the probability that each participant will complete a given wave of the NZAVS and is accurate at detecting whether participants will remain in the study (e.g. Time 13 *R* ^2^ = .985; Time 14 *R* ^2^ = .992; Time 15 *R* ^2^ = .987). Thus, we can assess whether participants in distinct classes are more (or less) likely to remain participants in the study.

## RESULTS

### Analytic approach

The present study examines whether distinct subgroups of the population differ in their trajectories of IRD and GRD over 12 years. Accordingly, we utilize LCGA in Mplus *v*.8.11 (Muthén & Muthén, 1998–2024) with full‐information maximum likelihood estimation (FIML; Enders & Bandalos, 2001). While traditional latent growth curve modelling assumes individuals share a general latent growth trajectory, LCGA allows for the possibility that different *groups* of individuals differ in their rates of change over time (Muthén & Muthén, 2000). This approach allows us to detect whether subgroups of the population are, for example, increasing in their feelings of relative deprivation while others may be relatively stable or decreasing over time.

In this study, we estimate multiple‐trajectory classes based on the trajectories of IRD *and* GRD, allowing us to examine potential combinations of these two forms of deprivation (see Runciman, 1966). As such, we estimated intercepts and rates of change for IRD and GRD in each class. Because prior work suggests relative deprivation exhibits curvilinear change over time (e.g. Lilly et al., 2025), we estimated both linear and quadratic rates of change for each class. We then freely estimated—but constrained across classes—the variances of the intercepts. Additionally, we constrained the residual variances across both classes and assessment occasions. Finally, because LCGA assumes that individuals within each class are homogenous with respect to their trajectories over time, we constrained the growth variances and covariances to 0 (see Muthén, 2004). Thus, individual differences were assumed to exist *between*—but not within—estimated classes.

### Model estimation and selection

We first estimated a model with a one‐class trajectory as a baseline for model comparisons. We then estimated models fitting between 2 and 5 classes to identify the model that best fit these data. We determined the best‐fitting model based on the Akaike Information Criterion (AIC), the Bayesian Information Criterion (BIC) and the sample‐size adjusted BIC (aBIC), with lower values indicating relatively better model fit (Muthén, 2004; Muthén & Muthén, 2000). We also inspected each model's classification precision (i.e. entropy), with values closer to 1.0 indicating a more precise separation of the data into distinct classes (Collins & Lanza, 2010).

Table 2 displays the model comparisons and indicates that the AIC, BIC and aBIC values reduced with the addition of each subsequent class. That said, relative improvements to model fit plateaued after the addition of a third class. Additionally, entropy decreased substantially to .76 in the three‐class model and continued to decline upon the addition of subsequent classes. As such, we identified the two‐class trajectory as the most parsimonious and best‐fitting model. Further supporting this model, Table 3 displays the average probability that a participant belonged to a given class and reveals a high average likelihood that participants were correctly assigned to their given class (.90–.98), with only a small likelihood of misclassification.

**TABLE 2:** Comparison of fit indices for latent class growth models for individual‐ and group‐based relative deprivation over a 12‐year period.

Model	AIC	BIC	aBIC	ΔAIC	ΔBIC	ΔaBIC	Entropy	Trajectory class prevalence (%)				
								1.	2.	3.	4.	5.
1 Class	2,073,801.36	2,073,902.85	2,073,867.89	–	–	–	1.000	100.00	–	–	–	–
2 Classes	2,056,006.90	2,056,172.98	2,056,115.77	17,794.46	17,729.87	17,752.12	0.887	90.46	9.55	–	–	–
3 Classes	2,051,000.99	2,051,231.64	2,051,152.19	5005.92	4941.33	4963.58	0.756	70.07	7.06	22.87	–	–
4 Classes	2,046,898.50	2,047,193.74	2,047,092.04	4102.49	4037.91	4060.16	0.580	24.67	58.41	9.92	7.01	–
5 Classes	2,044,301.72	2,044,661.55	2,044,537.60	2596.77	2532.19	2554.43	0.590	6.94	60.27	21.51	3.86	7.42

Abbreviations: aBIC, sample‐size adjusted Bayesian Information Criterion; AIC, Akaike Information Criterion; BIC, Bayesian Information Criterion.

**TABLE 3:** Average latent class probabilities for most likely class membership (row) by latent class (column).

Class	*N*	%	1	2
1. Content	67,907	90.455	**0.976**	0.024
2. Deprived	7166	9.545	0.097	**0.903**

*Note*: Values highlighted in bold reflect the average probability that a person estimated to belong to a given latent class was correctly categorized.

Finally, we replicated our models using two random split halves of our sample to validate the selected model (see Tables S1 and S2, Figure S1 in the Online Supporting Information). The two‐class solution was stable across the two samples and produced estimates analogous to our primary analysis. We are thus confident in the two‐class solution as the best fitting model for our data.

### Class trajectories

**FIGURE 1:**
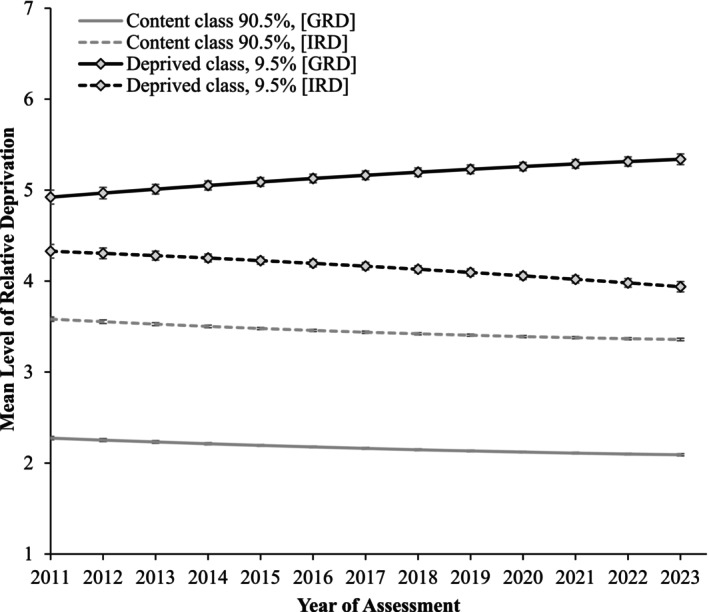
Multi‐trajectory latent class growth analysis of individual‐based relative deprivation (IRD) and group‐based relative deprivation (GRD) over 12 years. Error bars represent 95% confidence intervals.

Table 4 displays the parameter estimates for each latent class trajectory in the two‐class solution. Class 1, the *Content* class (see Figure 1), contained most of the sample (90.5%) and had low‐to‐moderate levels of IRD and low levels of GRD across the 13 assessment occasions (i.e. both constructs were below the mid‐point of the scale at each assessment). That said, the *Content* class demonstrated linear decreases in both IRD (*s* = −0.22, *SE* = 0.02, *p* < .001; *q* = 0.09, *SE* = 0.02, *p* < .001) and GRD (*s* = −0.18, *SE* = 0.01, *p* < .001; *q* = 0.06, *SE* = 0.02, *p* < .001) that slowed across the 12‐year period.

**TABLE 4:** Parameter estimates of the two‐class latent growth model.

Class	Group‐based relative deprivation						Individual‐based relative deprivation					
	Estimate	*SE*	95% CI		*p*‐value	Variance	Estimate	*SE*	95% CI		*p*‐value	Variance
			LB	UB					LB	UB		
1. Content												
*i*	2.19***	0.01	2.184	2.204	<.001	0.53***	3.48***	0.01	3.467	3.492	<.001	1.39***
*s*	−0.18***	0.01	−0.199	−0.153	<.001	0.00	−0.22***	0.02	−0.253	−0.191	<.001	0.00
*q*	0.06***	0.02	0.026	0.091	<.001	0.00	0.09***	0.02	0.046	0.130	<.001	0.00
2. Deprived												
*i*	5.09***	0.02	5.043	5.137	<.001	0.53***	4.23***	0.02	4.186	4.265	<.001	1.39***
*s*	0.38***	0.06	0.275	0.491	<.001	0.00	−0.29***	0.06	−0.399	−0.184	<.001	0.00
*q*	−0.09	0.08	−0.251	0.072	.279	0.00	−0.09	0.08	−0.241	0.071	.285	0.00

*Note*: Variances are constrained to equality across latent classes.

Abbreviations: 95% CI, confidence interval; *i*, intercept; LB, lower bound; *q*, quadratic slope; *s*, linear slope; UB, upper bound.

*** *p* < .001.

Class 2, the *Deprived* class, contained the remaining 9.5% of the sample and demonstrated consistently higher mean levels of IRD and GRD over time than the *Content* class; however, the difference was more pronounced for GRD (see Figure 1). Moreover, the *Deprived* class demonstrated significant linear decreases in IRD (*s* = −0.29, *SE* = 0.06, *p* < .001; *q* = −0.09, *SE* = 0.08, *p* = .285) but linear *increases* in GRD (*s* = 0.38, *SE* = 0.06, *p* < .001; *q* = −0.09, *SE* = 0.08, *p* = .279). Notably, equality tests of the trajectories for IRD (*Wald*_(3)_ = 1434.54, *p* < .001) and GRD (*Wald*_(3)_ = 26342.57, *p* < .001) revealed significant differences between the *Content* and *Deprived* classes. Thus, the *Deprived* class significantly differed from the *Content* class in its levels of relative deprivation over time.

### Demographic differences

We next conducted a three‐step multinomial logistic regression predicting class membership as a function of all the demographic covariates simultaneously using the *Content* class trajectory as the reference class. As shown in Table 5, the odds of belonging to the *Deprived* (versus *Content*) trajectory class were lower for those who owned their own home (*b* = −0.28, *SE* = 0.06, OR = 0.76, *p* < .001) and people born in New Zealand (*b* = −0.27, *SE* = 0.09, OR = 0.77, *p* = .002). Additionally, the odds of belonging to the *Deprived* (vs. *Content*) class were lower for older participants (*b* = −0.01, *SE* = 0.00, OR = 0.99, *p* = .002) and people with higher (vs. lower) annual household incomes (*b* = −0.06, *SE* = 0.03, OR = 0.94, *p* = .023).

**TABLE 5:** Multinomial logistic regression predicting the likelihood of belonging to the Deprived class (relative to the Content class) as a function of demographic covariates.

Predictor	Deprived (vs. content)							
	*B*	SE	95% CI		OR	95% CI		*p*
			LB	UB		LB	UB	
Gender^a^	−0.11	0.06	−0.218	0.008	0.90	0.804	1.008	.068
Māori^b^	5.08	0.11	4.870	5.287	160.56	130.369	197.734	<.001
Pacific^b^	4.85	0.13	4.601	5.100	127.80	99.598	163.980	<.001
Asian^b^	2.96	0.12	2.720	3.202	19.31	15.176	24.580	<.001
Age^c^	−0.01	0.00	−0.010	−0.002	0.99	0.990	0.998	.002
Born in NZ^d^	−0.27	0.09	−0.439	−0.097	0.77	0.645	0.907	.002
Homeowner^d^	−0.28	0.06	−0.399	−0.155	0.76	0.671	0.857	<.001
HH Income	−0.06	0.03	−0.118	−0.009	0.94	0.889	0.991	.023
Education	0.00	0.01	−0.023	0.019	1.00	0.977	1.019	.853
Ethnic ID	0.84	0.02	0.794	0.875	2.30	2.213	2.399	<.001

*Note*: HH Income = Household income in NZD$100,000.

^a^ Dummy‐coded (0 = woman, 1 = man).

^b^ Dummy‐coded (0 = No/New Zealand European, 1 = Yes).

^c^ Age at Time 3 (2011).

^d^ Dummy‐coded (0 = no, 1 = yes).

Conversely, the odds of belonging to the *Deprived* (vs. *Content*) trajectory class were higher for participants who identified as Māori (*b* = 5.08, *SE* = 0.11, OR = 160.56, *p* < .001), Pasifika (*b* = 4.85, *SE* = 0.13, OR = 127.80, *p* < .001) and Asian (*b* = 2.96, *SE* = 0.12, OR = 19.31, *p* < .001) relative to their European counterparts. Moreover, participants with higher levels of ethnic identification were more likely to belong to the *Deprived* (vs. *Content*) class (*b* = 0.84, *SE* = 0.02, OR = 2.30, *p* < .001). The odds of belonging to these two trajectory classes did not, however, reliably vary by gender (*b* = −0.11, *SE* = 0.06, OR = 0.90, *p* = .068) or education (*b* = 0.00, *SE* = 0.01, OR = 1.00, *p* = .853).

### Well‐being and sociopolitical attitudes

**FIGURE 2:**
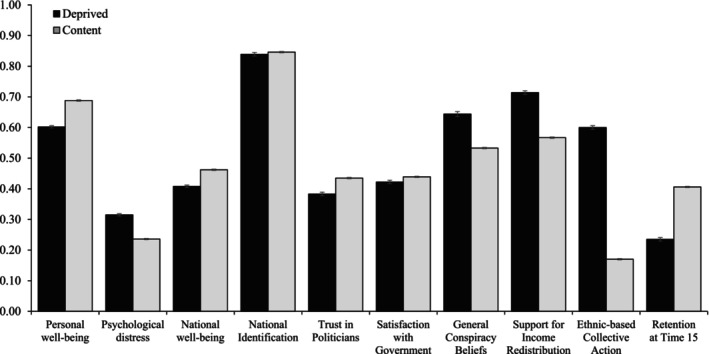
Mean well‐being and sociopolitical outcomes across the content and deprived classes. Measures were rescaled on a 0 to 1 scale to facilitate comparisons. Error bars represent 95% confidence intervals.

As a final validation of our two classes, we examined whether the *Deprived* and *Content* classes differed in their mean levels of key well‐being and sociopolitical outcomes using the distal three‐step procedure (Asparouhov & Muthén, 2014). To facilitate comparisons, we rescaled each measure to a 0 to 1 scale. Figure 2 shows that the *Deprived* and *Content* classes demonstrated consistent differences across all variables of interest (*p*s ≤ .016; see Table 6). Turning first to individual well‐being, participants in the *Deprived* class reported lower levels of personal well‐being and higher levels of psychological distress than the *Content* class. Additionally, participants in the Deprived class reported a lower sense of *national* well‐being than the *Content* class. Our results also suggest a marginal difference in national identification between the *Deprived* and *Content* classes (Δ*χ* ^2^_(1)_ = 5.84, *p* = .016).

**TABLE 6:** Mean scores on well‐being, sociopolitical attitudes and study retention across classes.

Variables	Deprived		Content		Chi‐square difference test		
	Mean	*SE*	Mean	*SE*	Δ*χ* ^2^	*df*	*p*‐value
Personal well‐being	0.60	0.002	0.69	0.001	1502.55	1	<.001
Psychological distress	0.32	0.002	0.24	0.001	1078.83	1	<.001
National well‐being	0.41	0.002	0.46	0.001	663.76	1	<.001
National identification	0.84	0.003	0.85	0.001	5.84	1	.016
Trust in politicians	0.38	0.003	0.44	0.001	254.59	1	<.001
Satisfaction with government	0.42	0.003	0.44	0.001	24.92	1	<.001
General conspiracy beliefs	0.64	0.004	0.53	0.001	877.74	1	<.001
Support for income redistribution	0.71	0.003	0.57	0.001	1606.28	1	<.001
Ethnic‐based collective action	0.60	0.003	0.17	0.001	21664.75	1	<.001
Retention at Time 15	0.24	0.003	0.41	0.001	3074.30	1	<.001

*Note*: Measures rescaled on a 0 (low) to 1 (high) scale to facilitate comparisons.

The *Deprived* class also reported lower levels of trust in politicians and government satisfaction than the *Content* class. Conversely, participants in the *Deprived* class reported higher levels of general conspiracy beliefs compared to the *Content* class. The *Deprived* class also reported more support than the *Content* class for income redistribution and collective action (see Figure 2). Finally, the *Deprived* class was significantly less likely to complete Time 15 than the *Content* class.

### Robustness checks

#### Affective relative deprivation

Although both cognitive and affective components are central to relative deprivation (for discussion, see Smith et al., 2012), our cognitive IRD item, ‘*I generally earn less than other people in New Zealand*’, may more closely reflect subjective socioeconomic status (e.g. see Callan et al., 2015). As such, we replicated our analyses focusing solely on our affective items (see the OSM for the full results). These analyses largely replicate our main results, albeit with some nuances. First, the *Deprived* class in the affect‐only model was slightly larger (12.0%) and displayed an increase in affective GRD that *slowed over time* (see Table S2 and Figure S2). More broadly, curvilinear changes were more pronounced for both IRD and GRD trajectories across classes (compared to our main analysis), suggesting that the affective measures may be more malleable than their cognitive counterparts.

Second, women and people higher (vs. lower) in education were more likely to belong to the *Deprived* (versus *Content*) class (these same effects were non‐significant in our main analysis; see Table S3). Additionally, while people born in New Zealand were *less* likely to belong to the *Deprived* class in our primary model, people born in New Zealand were *more* likely to belong to the *Deprived* class in our affect‐only analysis. Migrant status may thus be a less reliable predictor of class membership, or people born overseas may be more likely to feel disadvantaged but less likely to feel *frustrated* about their disadvantaged status. Nonetheless, the remaining predictors and mean differences across well‐being, sociopolitical and retention measures replicated our main results (Tables S3 and S4).

#### Income and relative deprivation

Studies suggest that material resources (or lack thereof) impact relative deprivation—particularly IRD (e.g. Lilly et al., 2024a; Smith et al., 2020). Our multinomial logistic regression supports this possibility, as people with lower incomes were more likely to belong to the *Deprived* (versus *Content*) class. However, income may also (partly) motivate *trajectories* of relative deprivation. We assessed this possibility by regressing the intercept and slope onto household income. To allow for classes to differ in their sensitivity to income, the effects of income were free to vary between classes.

Figure S3 and Table S5 display the results of these analyses. First, our analysis still identified two classes analogous to our primary analysis, though the differences between IRD trajectories across classes were smaller (see Figure S3). Table S5 displays the regression coefficients and reveals the distinct effects of income across classes. Namely, while participants with higher household incomes displayed lower initial scores of both IRD and GRD among the *Content* and *Deprived* classes, the effects of income were strongest on the intercept for IRD in the *Content* class. Additionally, higher income individuals in the *Content* class experienced a slower decline in relative deprivation over time, likely due to a floor effect, as lower initial IRD and GRD scores leave less room for ‘improvement’ over time. Conversely, income did not significantly affect the rates of change in the *Deprived* class, suggesting that income has a lesser impact on this class.

## DISCUSSION

Although Runciman (1966) presented four potential ‘types’ of relative deprivation, few studies examine this typology (although see Foster & Matheson, 1995; Osborne et al., 2015) and no studies to date examine how different subgroups perceive their relative status longitudinally. The current study addressed these oversights by examining trajectories of IRD and GRD simultaneously across 12 years of a nationwide random sample of adults. By utilizing LCGA, we investigated whether different subgroups of the population differ in their perceptions of IRD and GRD across time. This approach allowed us to directly test whether a proportion of the population experiences high levels of both IRD and GRD *simultaneously* (i.e. ‘double deprivation’, see Runciman, 1966) and whether distinct trajectories of IRD and GRD emerged over a 12‐year period (i.e. a personal/group discrepancy, see Crosby, 1984; Taylor et al., 1990).

We identified a *Content* class comprised of most of the population (90.5%) who scored relatively low on IRD and GRD, with evidence of declines in both constructs over time. These results corroborate previous assertions that most people do not perceive themselves as deprived relative to other individuals or groups (Osborne et al., 2015) and are generally ‘content’ with their societal position. Our results did, however, uncover a subset of the population (9.5%) who reported moderate levels of IRD and high levels of GRD across time. Critically, this *Deprived* class demonstrated *declines* in IRD but linear *increases* in GRD simultaneously, suggesting that this subgroup perceived themselves as increasingly collectively—but not personally—deprived. Perhaps more importantly, neither of these classes reported high or increasing levels of both IRD and GRD across time, suggesting that Runciman's ‘doubly‐deprived’ type is trivial or absent from this population.

### Demographic differences

We also identified demographic differences between the *Content* and *Deprived* classes. Specifically, the odds of belonging to the *Content* class were higher for Europeans, homeowners and, to a small extent, people who were older or had higher household incomes. These results support the assertion that individuals with *objective* societal advantages are less likely to perceive themselves as *relatively* deprived. Indeed, one would expect Europeans to report lower levels of relative deprivation due to their structural advantages vis‐à‐vis ethnic minorities. Likewise, those with objective material advantages (i.e. higher income and home ownership) are less likely to perceive themselves as relatively deprived (e.g. Foye et al., 2018; Liu et al., 2022). It is thus unsurprising that those who are objectively ‘well‐off’ were less likely to perceive themselves as unjustly disadvantaged.

### Well‐being and sociopolitical attitudes

Finally, we identified differences in well‐being, trust and satisfaction with institutions, and support for social change between classes. First, the *Deprived* class reported higher mean levels of psychological distress and lower mean levels of personal well‐being than the *Content* class, corroborating previous assertions that greater feelings of relative deprivation correlate with adverse well‐being outcomes (de la Sablonnière et al., 2010; Lilly et al., 2023; Smith et al., 2012; Smith et al., 2020). Moreover, the *Deprived* class reported lower levels of national well‐being, national identification, trust in politicians and government satisfaction, and higher levels of general conspiracy theory belief than the *Content* class. These results demonstrate the costs of perceived inequality and injustice, not only for individuals but for social cohesion and democracy (see Osborne et al., 2022), such that people's feelings of relative deprivation correlate with reduced trust in and satisfaction with existing social structures and institutions.

Our results also demonstrate that the probability of retaining participants in Time 15 was lower in the *Deprived* (vs. *Content*) class, suggesting that those who feel increasingly deprived may be less likely to continue participating in research than those who feel relatively content. This may partly be due to a general distrust in institutions and science among those who feel collectively disadvantaged (see Wilkinson & Pickett, 2010) or because participants in less stable economic situations are more likely to drop out of longitudinal research (Greaves et al., 2020; Satherley et al., 2015). The lower probability of retention among the *Deprived* class also suggests we may be *underestimating* the extent to which people feel deprived in New Zealand. While retention analyses are not the focus of the present study, identifying who does and does not respond to surveys is important for understanding who we are capturing in the population when assessing feelings of relative deprivation over time (for a discussion, see Greaves et al., 2020).

Finally, the *Deprived* class reported higher support for income redistribution and collective action than the *Content* class, which has implications for understanding how the public perceives and responds to inequality. While our results reveal that feeling relatively content improves one's well‐being (see also Osborne & Sibley, 2013; Schmitt et al., 2010), feeling *frustrated* about one's status is an integral antecedent to collective responses to inequality (e.g. see Abrams & Grant, 2012; Osborne et al., 2019; Smith et al., 2012). Our results suggest that most of the population has *not* felt this frustration over the last 12 years, which may help explain why we have not seen sustained responses to inequality (see Jost et al., 2017) and why some people *support* the upward transfer of wealth (Bartels, 2005). Indeed, despite growing disparities between the rich and the poor (Rashbrooke, 2013; Stock, 2023), sustained responses to income inequality in New Zealand are rare (with the exception of Occupy Aotearoa, 2011; see Hager, 2021). That only a small subgroup of the population reported high—and increasing—levels of GRD across time may explain why large‐scale action is so rare. Nonetheless, future research is needed to assess how distinct subgroups in the population respond to the continual perceived pressures (and lack thereof) of economic inequality.

### Implications for relative deprivation theory

Our results have important implications for relative deprivation theory. First, although we identify two classes that broadly correspond to Runciman's (1966) ‘orthodox’ (*Content*) and ‘fraternalist’ (*Deprived*) types, the absence of a ‘doubly‐deprived’ type suggests a need to reconsider Runciman's typology—and the relationship between IRD and GRD (see also Osborne et al., 2015). Indeed, rather than identifying a ‘doubly‐deprived’ class, our results corroborate and extend research on the personal/group discrimination discrepancy (see Crosby, 1984; Taylor et al., 1990). Specifically, as members of the *Deprived* class increasingly recognized their ethnic groups' disadvantages relative to other groups in New Zealand, their feelings of *personal* disadvantage declined. This discrepancy may emerge because group experiences of discrimination or disadvantage are more salient than personal experiences (e.g. Moghaddam et al., 1997) or because people perceive their groups as more deprived when making comparisons to dominant groups (Dumont et al., 2006; Tropp & Wright, 1999). Supporting this possibility, members of the *Deprived* class (relative to the *Content* class) were significantly more likely to be Māori, Pasifika or Asian than European, and were more likely to strongly identify with their ethnicity. Thus, membership in—and identification with—a structurally disadvantaged group may elicit this personal/group discrepancy (for a recent discussion, see McAvay & Safi, 2025). Nonetheless, future work is needed to determine whether this discrepancy generalizes across comparison targets beyond ethnicity (e.g. gender). Our results thus provide the foundation for future work studying how and when IRD and GRD may (or may not) emerge in tandem.

Second, the absence of a class high in *IRD* (i.e. the ‘strivers’; Runciman, 1966) suggests a need to evaluate when feelings of IRD may ‘outweigh’ GRD. Indeed, while the personal/group discrepancy explains the absence of the ‘doubly‐deprived’ class, this neither explains why a subgroup of people experiencing IRD did not emerge, nor why class differences were more pronounced for GRD than IRD. One possibility is that, in addition to changing socio‐economic conditions, changing societal *discussions* of group inequalities impacted relative deprivation trajectories. Indeed, social movements such as the Ihumātao occupation in New Zealand (2016–2020; Fernandes, 2019; Hancock & Newton, 2022) and the international Black Lives Matter movement (2016–present; Szetela, 2020) have brought increased attention to ethnic inequalities across domains, changing public perceptions of inequality (see Dunivin et al., 2022). Additionally, recent research suggests that *media* frames ethnic inequalities as illegitimate differences between groups, while broader wealth inequalities are seemingly more legitimate and typically described without a referent group (Jun et al., 2022). The absence of individual comparisons in inequality discussions may explain why a ‘high IRD’ class did not emerge and why GRD largely drives differences between classes. While beyond the scope of our data, future research could investigate how different framings of race and wealth influence feelings of relative deprivation to determine the conditions in which a ‘Strivers’ class may emerge.

Finally, our results highlight the need for longitudinal studies of relative deprivation. Most of the relative deprivation literature is cross‐sectional (see Smith et al., 2012), and the few longitudinal studies rarely examine how relative deprivation changes and develops over time (for an exception, see Lilly et al., 2025). Our study demonstrates the value of studying relative deprivation over periods of socio‐economic change (see de la Sablonnière, Tougas, et al., 2009; Osborne et al., 2019). More specifically, we highlight the importance of *person‐centred* longitudinal methods to relative deprivation research. Indeed, our use of LCGA—coupled with a large, nationwide random sample of adults and 12 years of data—identified two theoretically meaningful subgroups of the population that demonstrated distinct feelings of relative deprivation across time. That these two classes qualitatively differed in their demographic characteristics, well‐being and sociopolitical attitudes highlights the strengths of LCGA in identifying unique subgroups in the population (see Thomas et al., 2024). We thus encourage future work investigating typologies of change in relative deprivation over time across socio‐economic contexts to further understand how and when people respond to their objective conditions.

### Caveats and future directions

Despite the current study's important theoretical and practical implications, there are limitations worth noting. First, we caution against interpreting our results as a ‘*true*’ number of profiles or that specific demographic characteristics invariably predict profile membership. Instead, our analyses identify the *most likely* profile membership based on one's demographic predictors. These demographic predictors—and the number of profiles that emerge—may differ in other contexts. Indeed, while certainly experiencing similar socio‐economic crises to other (Western) nations, New Zealand is one of the most WEIRD and egalitarian nations in the world, which may limit the generalizability of our results. For example, the ‘doubly‐deprived’ profile (among others) may emerge in countries with greater levels of income inequality, fewer anti‐discrimination laws or less social welfare. These factors will likewise impact the stability and trajectories of relative deprivation over time. Accordingly, future research should examine the trajectories of IRD and GRD over time in non‐Western and less egalitarian contexts.

It is also important to note that we focus solely on fiscal forms of relative deprivation. Indeed, other forms of relative deprivation (e.g. those that focus on interpersonal treatment or discrimination) may develop and change differently across time, or a different number of subgroups may emerge in the population. While different forms of relative deprivation operate similarly (see Smith et al., 2012), future research should consider how and for whom other forms of relative deprivation change over time.

Finally, the current study neither disentangles the *causal* processes underlying changes in relative deprivation, nor rules out unmeasured socio‐economic and structural factors affecting our results. That said, our study examines relative deprivation in a general population sample over a period of considerable socioeconomic change—a valuable approach to studying real‐world phenomena (Diener et al., 2022). We thus encourage methodological pluralism in studies of relative deprivation, and future work should use both observational and experimental approaches to study changes in perceived disadvantage over time. Relatedly, while we assessed the sociopolitical attitudes of our distinct profiles, we did not assess whether the classes were *changing* in their attitudes distinctly over time. For example, while we identified that the *Deprived* (versus the *Content*) class reported stronger collective action support, we could not assess whether this was due to the subgroup *increasing* their support over time as a function of feelings of relative deprivation. More broadly, if a small subgroup of the population perceives themselves as increasingly collectively deprived, this may have significant consequences for potential radicalization and extremism (e.g. see Kunst & Obaidi, 2020; Osborne et al., 2025). Future research should consider this possibility by assessing whether different change trajectories in relative deprivation, in turn, predict distinct change trajectories in constructs central to relative deprivation theory (i.e. psychological well‐being and collective action support).

## CONCLUSION

Despite over 75 years of research, few studies have examined ‘types’ of relative deprivation, nor how they may manifest longitudinally. The present study addressed these oversights using multi‐trajectory LCGA to identify distinct subgroups in the population that differ in their rates of change in IRD and GRD over 12 years. Our results revealed that most of the population was relatively content over time, with low scores on IRD and GRD that declined over time. Conversely, the *Deprived* class demonstrated moderate scores on IRD that decreased, but *high* scores on GRD that *increased* over time. These two classes differed across key demographics, as well as in well‐being and sociopolitical attitudes, revealing a considerable divide between the ‘haves’ and the ‘have‐nots’ in New Zealand. Overall, our results provide a more nuanced understanding of *how* people feel deprived, as well as a springboard for future research examining typologies of relative deprivation over time.

## AUTHOR CONTRIBUTIONS

**Kieren J. Lilly:** Conceptualization; methodology; formal analysis; writing – review and editing; writing – original draft. **Chris G. Sibley:** Data curation; supervision; funding acquisition; writing – review and editing. **Danny Osborne:** Supervision; writing – review and editing.

## CONFLICT OF INTEREST STATEMENT

The author(s) confirm that we have no potential conflicts of interest to declare and that the data described in this paper stem from research conducted in adherence to the APA Code of Conduct.

## Supporting information


Data S1.

## Data Availability

This study was not pre‐registered. The data described in the paper are part of the New Zealand Attitudes and Values Study (NZAVS). Full copies of the NZAVS data files are held by all members of the NZAVS management team and advisory board. A de‐identified dataset containing the variables analysed in this manuscript is available upon request from the corresponding author, or any member of the NZAVS advisory board for the purposes of replication or checking of any published study using NZAVS data. The Mplus syntax used to test all models reported in this manuscript is available on the Open Science Framework: https://osf.io/75snb/.

## Footnotes

^1^
Comparisons to others in a given nation do not necessarily provide enough proximity to measure IRD. However, New Zealand is a small nation with a labour force characterized by a ‘small‐world’ network—a sparse network with high amounts of clustering (e.g. see MacGibbon, 2008). That is, New Zealand is highly interconnected and ‘people in New Zealand’ is a meaningful comparison target in this context, although we acknowledge that these items may not be applicable in other nations.
