# Supplementary material for: The haves and the have‐nots: Identifying typologies of change in relative deprivation using multi‐trajectory latent class growth analysis
Source: Br J Soc Psychol. 2025 Aug 16;64(4):e70009. doi: 10.1111/bjso.70009 (PMC12357168; doi:10.1111/bjso.70009)
Supplement: Supplementary file 1 — Data S1. [file BJSO-64-0-s001.docx]

**The Haves and the Have-nots: Identifying Typologies of Change in Relative Deprivation using Multi-trajectory Latent Class Growth Analysis**

Online Supplementary Materials

**Table of Contents**

[Table S1. Comparison of fit indices for latent class growth models of individual- and group-based relative deprivation across robustness checks. 2](#_Toc198656857)

[Table S2. Parameter estimates for the Content and Deprived classes across robustness checks. 3](#_Toc198656858)

[Figure S1. Comparison of Content and Deprived class trajectories across two random halves of the sample. 4](#_Toc198656859)

[Figure S2. Latent class trajectories for the Content and Deprived classes using the affective relative deprivation items only. 5](#_Toc198656860)

[Table S3. Multinominal logistic regression predicting the likelihood of belonging to the (affective) Deprived class (relative to the Content class) as a function of demographic covariates. 6](#_Toc198656861)

[Table S4. Mean well-being, sociopolitical and retention scores across classes in the affective relative deprivation model. 7](#_Toc198656862)

[Figure S3. Latent class trajectories for the Content and Deprived classes, controlling for participants’ household income. 8](#_Toc198656863)

[Table S5. Regression coefficients for the effects of household income on trajectory intercepts and rates of change across classes. 9](#_Toc198656864)

## Table S1. *Comparison of fit indices for latent class growth models of individual- and group-based relative deprivation across robustness checks.*

|  |  |  |  |  |  |  |  | **Trajectory Class Prevalence** (%) | | | | |
| --- | --- | --- | --- | --- | --- | --- | --- | --- | --- | --- | --- | --- |
| Model | AIC | BIC | aBIC | ΔAIC | ΔBIC | ΔaBIC | Entropy | 1 | 2 | 3 | 4 | 5 |
| **Split A** (n = 37,454) | | | | | | | | | | | | |
| 1 Class | 1034842.74 | 1034936.58 | 1034901.62 | ---- | ---- | ---- | 1.000 | 100.0% | ---- | ---- | ---- | ---- |
| **2 Classes** | **1025965.76** | **1026119.32** | **1026062.11** | **8876.98** | **8817.26** | **8839.51** | **0.884** | **9.7%** | **90.3%** | ---- | ---- | ---- |
| 3 Classes | 1023502.14 | 1023715.41 | 1023635.96 | 2463.63 | 2403.91 | 2426.16 | 0.757 | 22.3% | 70.6% | 7.1% | ---- | ---- |
| 4 Classes | 1021234.49 | 1021507.47 | 1021405.78 | 2267.65 | 2207.94 | 2230.18 | 0.576 | 57.9% | 24.2% | 10.9% | 7.0% | ---- |
| 5 Classes | 1020034.53 | 1020367.23 | 1020243.29 | 1199.96 | 1140.24 | 1162.49 | 0.591 | 21.1% | 3.6% | 7.7% | 60.7% | 6.9% |
| **Split B** (n = 37,619) | | | | | | | | | | | | |
| 1 Class | 1038969.54 | 1039063.43 | 1039028.47 | ---- | ---- | ---- | 1.000 | 100.0% | ---- | ---- | ---- | ---- |
| **2 Classes** | **1030048.63** | **1030202.27** | **1030145.07** | **8920.90** | **8861.16** | **8883.40** | **0.889** | **9.4%** | **90.6%** | ---- | ---- | ---- |
| 3 Classes | 1027519.52 | 1027732.90 | 1027653.45 | 2529.12 | 2469.37 | 2491.62 | 0.755 | 23.4% | 7.0% | 69.6% | ---- | ---- |
| 4 Classes | 1025684.09 | 1025957.22 | 1025855.52 | 1835.43 | 1775.68 | 1797.93 | 0.583 | 7.0% | 9.3% | 25.0% | 58.7% | ---- |
| 5 Classes | 1024283.50 | 1024616.38 | 1024492.44 | 1400.59 | 1340.84 | 1363.09 | 0.594 | 22.8% | 7.0% | 2.9% | 59.0% | 8.3% |
| **Affect RD items only** (n = 74,316) | | | | | | | | | | | | |
| 1 Class | 2257652.18 | 2257753.56 | 2257718.60 | ---- | ---- | ---- | 1.000 |  | ---- | ---- | ---- | ---- |
| **2 Classes** | **2247067.02** | **2247232.91** | **2247175.71** | **10585.16** | **10520.65** | **10542.90** | **0.785** | **88.0%** | **12.0%** | ---- | ---- | ---- |
| 3 Classes | 2243682.70 | 2243913.10 | 2243833.65 | 3384.32 | 3319.81 | 3342.06 | 0.752 | 10.8% | 86.2% | 3.1% | ---- | ---- |
| 4 Classes | 2238464.13 | 2238759.04 | 2238657.34 | 5218.57 | 5154.06 | 5176.31 | 0.669 | 6.4% | 5.1% | 23.9% | 64.7% | ---- |
| 5 Classes | 2235149.89 | 2235509.32 | 2235385.38 | 3314.23 | 3249.72 | 3271.96 | 0.623 | 4.5% | 6.3% | 25.5% | 55.7% | 8.1% |

*Note.* Selected solution highlighted in bold. AIC = Akaike Information Criterion; BIC = Bayesian Information Criterion; aBIC = Sample-size adjusted Bayesian Information Criterion.

## Table S2. *Parameter estimates for the Content and Deprived classes across robustness checks.*

|  |  | **Group-based Relative Deprivation** | | | | | |  | **Individual-based Relative Deprivation** | | | | | |
| --- | --- | --- | --- | --- | --- | --- | --- | --- | --- | --- | --- | --- | --- | --- |
|  |  |  |  | **95% CI** | |  |  |  |  |  | **95% CI** | |  |  |
| Class |  | Est. | *SE* | LB | UB | *p*-value | Variance |  | Est. | *SE* | LB | UB | *p*-value | Variance |
| **Split A** |  |  |  |  |  |  |  |  |  |  |  |  |  |  |
| **1.      Content** | *i* | 2.19 | 0.01 | 2.174 | 2.203 | < .001 | 0.52^***^ |  | 3.47 | 0.01 | 3.456 | 3.491 | < .001 | 1.40^***^ |
|  | *s* | -0.16 | 0.02 | -0.197 | -0.132 | < .001 | 0.00 |  | -0.23 | 0.02 | -0.275 | -0.186 | < .001 | 0.00 |
|  | *q* | 0.04 | 0.02 | -0.011 | 0.081 | .140 | 0.00 |  | 0.09 | 0.03 | 0.035 | 0.153 | .002 | 0.00 |
|  |  |  |  |  |  |  |  |  |  |  |  |  |  |  |
| **2.      Deprived** | *i* | 5.06 | 0.03 | 4.995 | 5.128 | < .001 | 0.52^***^ |  | 4.25 | 0.03 | 4.190 | 4.302 | < .001 | 1.40^***^ |
|  | *s* | 0.26 | 0.08 | 0.110 | 0.406 | 0.001 | 0.00 |  | -0.33 | 0.08 | -0.481 | -0.175 | < .001 | 0.00 |
|  | *q* | 0.11 | 0.12 | -0.123 | 0.335 | .365 | 0.00 |  | -0.05 | 0.12 | -0.270 | 0.180 | .697 | 0.00 |
| **Split B** |  |  |  |  |  |  |  |  |  |  |  |  |  |  |
| **1.      Content** | *i* | 2.20 | 0.01 | 2.185 | 2.214 | < .001 | 0.53^***^ |  | 3.49 | 0.01 | 3.468 | 3.502 | < .001 | 1.38^***^ |
|  | *s* | -0.19 | 0.02 | -0.220 | -0.155 | < .001 | 0.00 |  | -0.21 | 0.02 | -0.257 | -0.170 | < .001 | 0.00 |
|  | *q* | 0.08 | 0.02 | 0.036 | 0.129 | .001 | 0.00 |  | 0.08 | 0.03 | 0.023 | 0.141 | .006 | 0.00 |
|  |  |  |  |  |  |  |  |  |  |  |  |  |  |  |
| **2.      Deprived** | *i* | 5.12 | 0.03 | 5.053 | 5.184 | < .001 | 0.53^***^ |  | 4.20 | 0.03 | 4.148 | 4.260 | < .001 | 1.38^***^ |
|  | *s* | 0.51 | 0.08 | 0.352 | 0.668 | < .001 | 0.00 |  | -0.26 | 0.08 | -0.405 | -0.106 | .001 | 0.00 |
|  | *q* | -0.29 | 0.12 | -0.518 | -0.063 | .012 | 0.00 |  | -0.13 | 0.11 | -0.341 | 0.087 | .244 | 0.00 |
| **Affect RD items only** |  |  |  |  |  |  |  |  |  |  |  |  |  |  |
| **1.      Content** | *i* | 2.25 | 0.01 | 2.231 | 2.268 | < .001 | 0.53^***^ |  | 3.27 | 0.01 | 3.250 | 3.282 | < .001 | 1.60^***^ |
|  | *s* | -0.18 | 0.02 | -0.215 | -0.154 | < .001 | 0.00 |  | -0.57 | 0.02 | -0.604 | -0.526 | < .001 | 0.00 |
|  | *q* | -0.10 | 0.02 | -0.147 | -0.056 | < .001 | 0.00 |  | 0.54 | 0.03 | 0.492 | 0.597 | < .001 | 0.00 |
|  |  |  |  |  |  |  |  |  |  |  |  |  |  |  |
| **2.      Deprived** | *i* | 4.84 | 0.04 | 4.774 | 4.912 | < .001 | 0.53^***^ |  | 4.12 | 0.02 | 4.078 | 4.167 | < .001 | 1.60^***^ |
|  | *s* | 0.76 | 0.08 | 0.605 | 0.912 | < .001 | 0.00 |  | -0.24 | 0.07 | -0.372 | -0.098 | .001 | 0.00 |
|  | *q* | -0.99 | 0.12 | -1.215 | -0.765 | < .001 | 0.00 |  | -0.23 | 0.10 | -0.416 | -0.042 | .017 | 0.00 |
| *Note.* Variances are constrained to equality across latent classes. 95% CI = Confidence Interval; LB = Lower Bound; UB = Upper Bound. *i* = intercept; *s* = linear slope; *q* = quadratic slope. ^***^*p* < .001. | | | | | | | | | | | | | | |

##
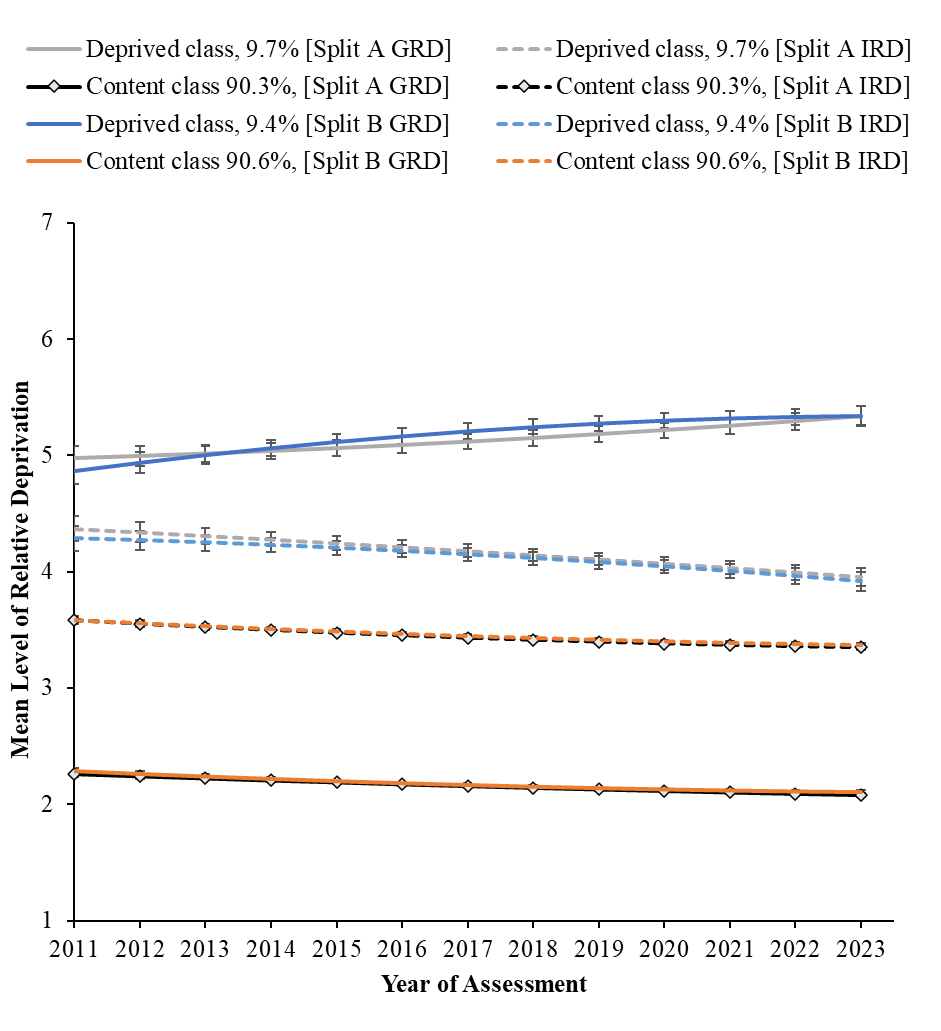
Figure S1. *Comparison of Content and Deprived class trajectories across two random halves of the sample.*

*Note.* Error bars represent 95% Confidence Intervals.

##
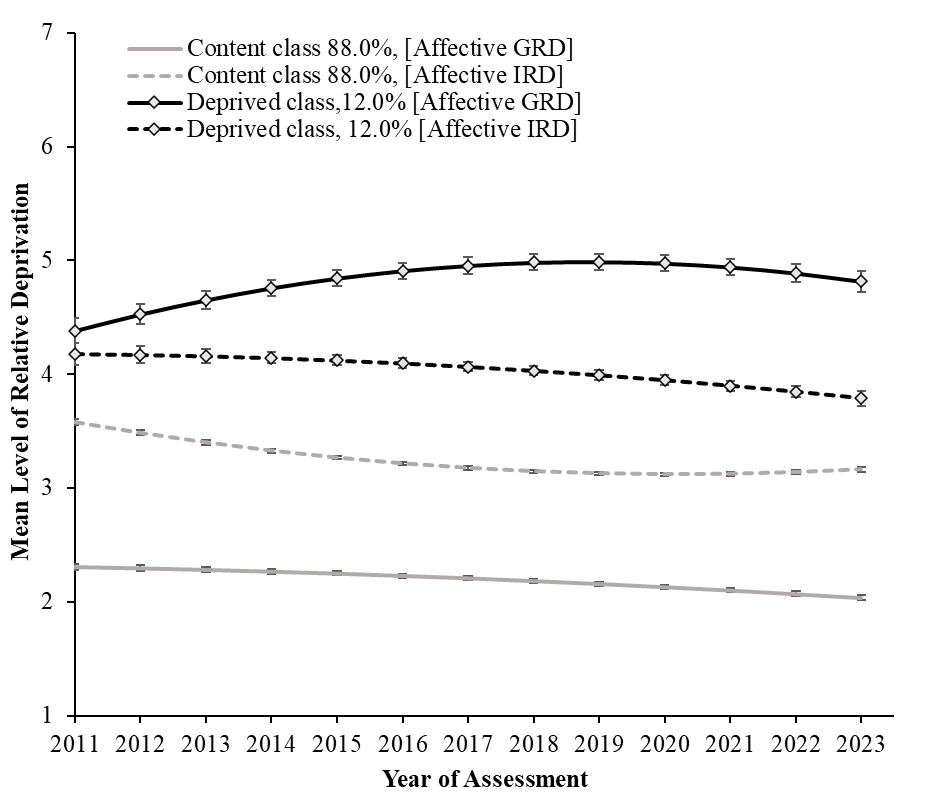
Figure S2. *Latent class trajectories for the Content and Deprived classes using the affective relative deprivation items only.*

*Note.* Error bars represent 95% Confidence Intervals.

## Table S3. *Multinominal logistic regression predicting the likelihood of belonging to the (affective) Deprived class (relative to the Content class) as a function of demographic covariates.*

|  | **Deprived** (versus Content) | | | | | | | |
| --- | --- | --- | --- | --- | --- | --- | --- | --- |
|  |  |  | 95% CI | |  | 95% CI | |  |
| Predictor | *B* | *SE* | LB | UB | OR | LB | UB | *p* |
| Gender^a^ | -0.19 | 0.05 | -0.289 | -0.098 | 0.82 | 0.749 | 0.907 | < .001 |
| Māori^b^ | 2.70 | 0.05 | 2.594 | 2.805 | 14.87 | 13.383 | 16.527 | < .001 |
| Pacific^b^ | 2.48 | 0.09 | 2.307 | 2.649 | 11.92 | 10.040 | 14.140 | < .001 |
| Asian^b^ | 1.05 | 0.09 | 0.880 | 1.217 | 2.85 | 2.411 | 3.377 | < .001 |
| Age^c^ | -0.01 | 0.00 | -0.016 | -0.009 | 0.99 | 0.984 | 0.991 | < .001 |
| Born in NZ^d^ | 0.37 | 0.07 | 0.245 | 0.501 | 1.45 | 1.278 | 1.650 | < .001 |
| Homeowner^d^ | -0.42 | 0.05 | -0.525 | -0.321 | 0.66 | 0.592 | 0.726 | < .001 |
| Household income | -0.14 | 0.03 | -0.194 | -0.075 | 0.87 | 0.824 | 0.927 | < .001 |
| Education | 0.03 | 0.01 | 0.015 | 0.049 | 1.03 | 1.015 | 1.050 | < .001 |
| Ethnic identification | 0.69 | 0.02 | 0.652 | 0.720 | 1.99 | 1.920 | 2.054 | < .001 |

*Note.* HH Income = Household income in NZD$100,000. ^a^Dummy-coded (0 = woman, 1 = man); ^b^Dummy-coded (0 = No/New Zealand European, 1 = Yes). ^c^Age at Time 3 (2011). ^d^Dummy-coded (0 = no, 1 = yes).

## Table S4. *Mean well-being, sociopolitical and retention scores across classes in the affective relative deprivation model.*

|  | **Deprived** | | **Content** | | **Chi-square difference test** | | | |
| --- | --- | --- | --- | --- | --- | --- | --- | --- |
| Variable | *Mean* | *SE* | *Mean* | *SE* | *Mdiff* | Δχ2 | *df* | *p*-value |
| Personal well-being | 0.599 | 0.002 | 0.695 | 0.001 | 0.096 | 2777.99 | 1 | < .001 |
| Psychological distress | 0.324 | 0.002 | 0.229 | 0.001 | -0.095 | 2077.98 | 1 | < .001 |
| National well-being | 0.396 | 0.002 | 0.466 | 0.001 | 0.070 | 1403.13 | 1 | < .001 |
| National identification | 0.833 | 0.002 | 0.847 | 0.001 | 0.014 | 29.05 | 1 | < .001 |
| Trust in politicians | 0.387 | 0.003 | 0.437 | 0.001 | 0.050 | 310.91 | 1 | < .001 |
| Satisfaction with government | 0.415 | 0.003 | 0.441 | 0.001 | 0.026 | 69.47 | 1 | < .001 |
| General conspiracy beliefs | 0.622 | 0.003 | 0.531 | 0.001 | -0.091 | 782.18 | 1 | < .001 |
| Support for income redistribution | 0.766 | 0.003 | 0.554 | 0.001 | -0.212 | 4912.13 | 1 | < .001 |
| Ethnic-based collective action | 0.493 | 0.003 | 0.164 | 0.001 | -0.329 | 15270.66 | 1 | < .001 |
| Retention at Time 15 | 0.269 | 0.003 | 0.410 | 0.001 | 0.141 | 2330.381 | 1 | < .001 |

*Note*. Measures were rescaled on a 0 (low) to 1 (high) scale to facilitate comparisons.

## Figure S3. *Latent class trajectories for the Content and Deprived classes, controlling for participants’ household income.*


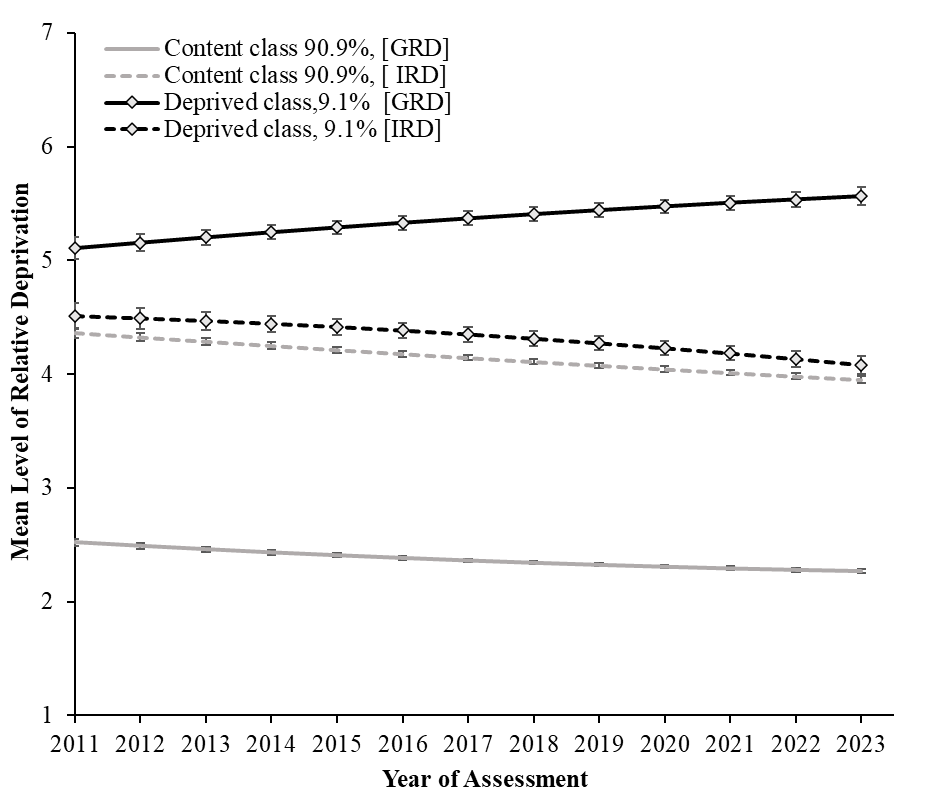


*Note.* Error bars represent 95% Confidence Intervals.

## Table S5. *Regression coefficients for the effects of household income on trajectory intercepts and rates of change across classes.*

|  | **Content class** | | | | |  | **Deprived class** | | | | |
| --- | --- | --- | --- | --- | --- | --- | --- | --- | --- | --- | --- |
|  |  |  | 95% CI | |  |  |  |  | 95% CI | |  |
| Parameter | *Est.* | *SE* | LB | UB | *p* |  | *Est.* | *SE* | LB | UB | *p* |
| **GRD** |  |  |  |  |  |  |  |  |  |  |  |
| *Intercept* | -0.21 | 0.01 | -0.222 | -0.198 | < .001 |  | -0.22 | 0.02 | -0.253 | -0.177 | < .001 |
| *Linear slope* | 0.08 | 0.02 | 0.051 | 0.112 | < .001 |  | 0.02 | 0.03 | -0.044 | 0.077 | .589 |
| *Quadratic slope* | -0.05 | 0.02 | -0.093 | -0.010 | .015 |  | -0.03 | 0.05 | -0.118 | 0.068 | .593 |
| **IRD** |  |  |  |  |  |  |  |  |  |  |  |
| *Intercept* | -0.71 | 0.01 | -0.734 | -0.689 | < .001 |  | -0.27 | 0.03 | -0.322 | -0.219 | < .001 |
| *Linear slope* | 0.17 | 0.02 | 0.131 | 0.217 | < .001 |  | 0.06 | 0.05 | -0.047 | 0.167 | .272 |
| *Quadratic slope* | 0.00 | 0.03 | -0.059 | 0.050 | .880 |  | 0.03 | 0.07 | -0.103 | 0.172 | .625 |

*Note.*  95% CI = Confidence Interval; LB = Lower Bound; UB = Upper Bound.
